# Prediction of antibiotic resistance mechanisms using a protein language model

**DOI:** 10.1093/bioinformatics/btae550

**Published:** 2024-09-10

**Authors:** Kanami Yagimoto, Shion Hosoda, Miwa Sato, Michiaki Hamada

**Affiliations:** Department of Electrical Engineering and Bioscience, Graduate School of Advanced Science and Engineering, Waseda University, Tokyo 169-8555, Japan; Center for Exploratory Research, Research and Development Group, Hitachi, Ltd, Tokyo 185-8601, Japan; Center for Exploratory Research, Research and Development Group, Hitachi, Ltd, Tokyo 185-8601, Japan; Department of Electrical Engineering and Bioscience, Graduate School of Advanced Science and Engineering, Waseda University, Tokyo 169-8555, Japan; Computational Bio Big-Data Open Innovation Laboratory (CBBD-OIL), National Institute of Advanced Industrial Science and Technology, Tokyo 169-8555, Japan; Graduate School of Medicine, Nippon Medical School, Tokyo 113-8602, Japan

## Abstract

**Motivation:**

Antibiotic resistance has emerged as a major global health threat, with an increasing number of bacterial infections becoming difficult to treat. Predicting the underlying resistance mechanisms of antibiotic resistance genes (ARGs) is crucial for understanding and combating this problem. However, existing methods struggle to accurately predict resistance mechanisms for ARGs with low similarity to known sequences and lack sufficient interpretability of the prediction models.

**Results:**

In this study, we present a novel approach for predicting ARG resistance mechanisms using ProteinBERT, a protein language model (pLM) based on deep learning. Our method outperforms state-of-the-art techniques on diverse ARG datasets, including those with low homology to the training data, highlighting its potential for predicting the resistance mechanisms of unknown ARGs. Attention analysis of the model reveals that it considers biologically relevant features, such as conserved amino acid residues and antibiotic target binding sites, when making predictions. These findings provide valuable insights into the molecular basis of antibiotic resistance and demonstrate the interpretability of pLMs, offering a new perspective on their application in bioinformatics.

**Availability and implementation:**

The source code is available for free at https://github.com/hmdlab/ARG-BERT. The output results of the model are published at https://waseda.box.com/v/ARG-BERT-suppl.

## 1 Introduction

The prevalence of antibiotic-resistant bacteria is increasing and poses a significant threat to global public health. If no action is taken, the annual number of deaths resulting from such infections is expected to reach 10 million by 2050 (O’Neill 2016). Thus, it is imperative to urgently address the spread of antibiotic resistance. Recent global and national action plans prioritize the One Health approach (World Health Organization *et al.* 2015), recognizing the relationships of human, animal, and environmental health. This is because the reservoirs of antibiotic resistance genes (ARGs) formed in various niches across these regions increase the damage of antibiotic resistance (Kim and Cha 2021). Therefore, it is necessary to investigate ARG reservoirs, which can be, e.g. identifying ARGs or predicting and analyzing resistant mechanisms (Alcock *et al.* 2023). Identifying ARGs in reservoirs is a crucial step in quantifying their presence and assessing the potential risk of antibiotic resistance. On the other hand, predicting and analyzing ARG resistance mechanisms in reservoirs are essential for selecting effective existing antibiotics and developing new antibiotics. Despite the large number of models for identifying ARGs in recent years (Gupta *et al.* 2014, Arango-Argoty *et al.* 2018, Feldgarden *et al.* 2021, Florensa *et al.* 2022, Alcock *et al.* 2023, Wu *et al.* 2023, Borelli *et al.* 2024), there are a few models for predicting and analyzing resistance mechanisms.

Current approaches for predicting resistance mechanisms can be categorized into two main types. The first and most common approach, exemplified by Comprehensive Antibiotic Resistance Database - Resistance Gene Identifier (CARD-RGI; Hendriksen et al. 2019, Alcock et al. 2023), relies on sequence homology search tools. These tools are employed to identify sequences similar to the input sequences from the ARG reference database. The advantage of these methods is a lower false-positive rate due to the similarity score cut-off. However, it is crucial to recognize that the cut-off similarity score restricts the applicability of these methods to ARGs with low similarity to sequences in the reference database, potentially leading to an increased false-negative rate (Arango-Argoty *et al.* 2018, Hendriksen *et al.* 2019, Li *et al.* 2021). The second approach involves the application of deep learning models, such as HMD-ARG and LM-ARG. HMD-ARG (Li *et al.* 2021) has employed a convolutional neural network (CNN), while LM-ARG (Ahmed *et al.* 2022) has used the embedding from protein language models (pLMs). Although these models can accurately predict sequences with low similarity to reference databases, they do not provide any interpretation of the predicted resistance mechanisms (Li *et al.* 2021). In summary, there is currently no resistance mechanism prediction method that achieves both scalability to sequences dissimilar to the reference database and interpretability of the results. Therefore, predictive models of resistance mechanisms incorporating these features are essential to tackle the challenge of antibiotic resistance.

Bidirectional Encoder Representations from Transformers (BERT; Devlin *et al.* 2019), a deep learning model in natural language processing, may offer accuracy and interpretability. BERT-based pLMs, pretrained on large protein datasets, have demonstrated improved performance and interpretability compared to existing deep learning models (Vig *et al.* 2020, Elnaggar *et al.* 2021, Brandes *et al.* 2022). The interpretability is attributed to attention, which identifies biological features contributing to the task (Vig *et al.* 2020, Brandes *et al.* 2022, Yamada and Hamada 2022, Lee *et al.* 2023, Zhou *et al.* 2023).

**Figure 1.**
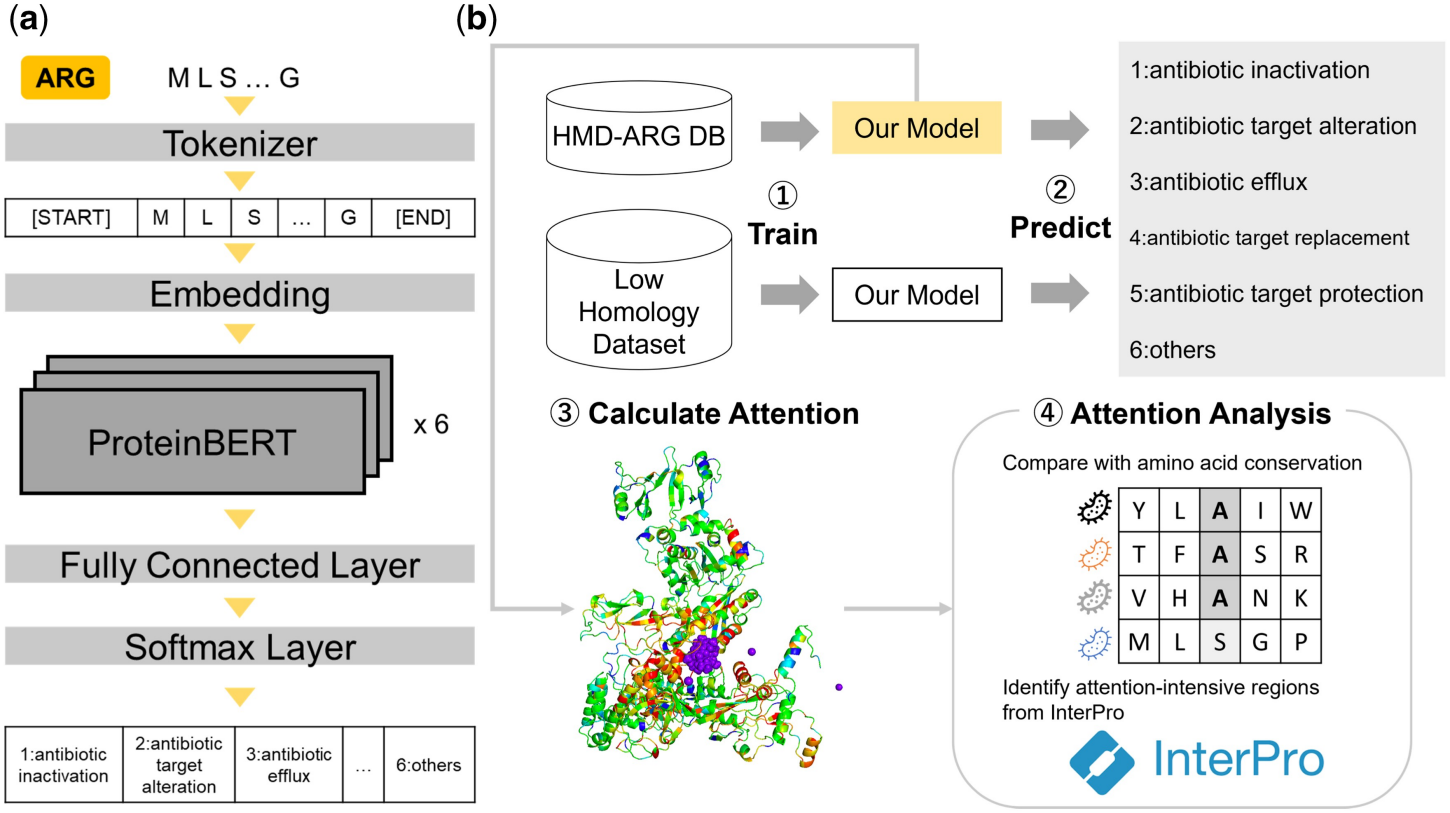
Overview of this study. (a) The architecture of the proposed model for predicting ARG resistance mechanisms. The model, based on ProteinBERT, a BERT-based pLM, includes an input layer for ARG sequences, and an output layer for predicting six resistance mechanism labels: antibiotic efflux, antibiotic inactivation, antibiotic target alteration, antibiotic target protection, antibiotic target replacement, and others. (b) The model was fine-tuned using the HMD-ARG DB and a custom-built low-homology dataset. Subsequently, attention analysis was performed on the fine-tuned model (with HMD-ARG DB) to interpret its predictions, suggesting that the proposed method takes into account biologically relevant features, such as antibiotic-binding sites and evolutionarily conserved regions, when making predictions.

In this study, we propose a novel method for predicting resistance mechanisms of ARGs using ProteinBERT (Brandes *et al.* 2022), a pLM pretrained on a large protein dataset (see Fig. 1 for an overview of this study). Our model performed as well as, or better than, existing models for ARG datasets containing sequences with low similarity to reference sequences. Through attention analysis, we demonstrated the interpretability of our model by identifying a correlation between attention and amino acid conservation and a concentration of attention on target binding sites and conserved regions involved in antibiotic resistance. Our proposed approach represents a promising strategy for mitigating the adverse effects of antibiotic resistance.

## 2 Materials and methods

### 2.1 Dataset collection

In this study, two datasets were utilized to fine-tune and evaluate the model as follows.

#### 2.1.1 HMD-ARG DB

The first dataset, the HMD-ARG DB, was created by Li *et al.* (2021) and is currently the largest available database, integrating seven publicly available ARG databases. The DNA sequences were converted to protein sequences, and duplicate sequences were removed before merging the seven ARG databases, resulting in a total of 24 082 ARGs. Each ARG was then annotated with labels corresponding to antibiotic efflux, antibiotic inactivation, antibiotic target alteration, antibiotic target protection, antibiotic target replacement, and other resistance mechanisms. The annotation of resistance mechanism labels was based on the Comprehensive Antibiotic Resistance Database (CARD; Alcock *et al.* 2023) ontology, using the Basic Local Alignment Search Tool (BLASTP; Altschul *et al.* 1990) or literature review. The complete dataset, along with the number of sequences corresponding to each resistance mechanism, is presented in Supplementary Fig. S1. See also the previous paper (Li *et al.* 2021) for details of this dataset.

To assess the performance using the datasets, we employed *stratified* 5-fold cross-validation (CV). Each dataset was divided into five separate groups, ensuring that the proportion of resistance mechanisms in each group was equal.

#### 2.1.2 Low homology dataset

To evaluate the performance of the proposed method on sequences with low similarity to known ARGs, we created a second dataset from the HMD-ARG DB (Section 2.1.1), named the “Low Homology Dataset,” which features reduced homology between training and test data. First, we employed the tool Cluster Database at High Identity with Tolerance(CD-HIT; Fu *et al.* 2012) to cluster sequences with the same resistance mechanism and sequence identity greater than 0.4. Each cluster is regarded as an identical group. We divided the unclustered dataset into five groups and added sequences assigned to 5-folds. In our evaluation, we used the Group K-fold package in Scikit-learn (Pedregosa *et al.* 2011) for the clusters and mechanisms. This approach resulted in a maximum sequence identity of 0.4 between training and test data for sequences with the same resistance mechanism. The same procedure was then applied to create datasets with sequence identity thresholds of 0.5, 0.6, 0.7, 0.8, and 0.9.

### 2.2 A pretrained pLM

In this study, we employed ProteinBERT, a type of pLM pretrained on protein sequences and Gene Ontology (GO) terms (Ashburner *et al.* 2000, Gene Ontology Consortium *et al.* 2023) that define the biological functions of genes. ProteinBERT consists of two components: one that processes GO terms as a *global* representation, $x\in R^{d_{\mathrm{global}}}$ ($d_{\mathrm{global}}=128$), and another that processes amino acid sequence data as a *local* representation $\left\{s_{i}\}(s_{i}\in R^{d_{\mathrm{local}}}\right)$, where *i* shows the position in a protein sequence. The global and local representations are connected via global attention (Section 2.6). In the pretraining step, the architecture was trained on 106 million protein sequences from UniProtKB/UniRef90 (Suzek *et al.* 2007) and 9000 GO terms by applying the same Masked Language Modeling (MLM) method as BERT. See Brandes *et al.* (2022) for the detailed model of ProteinBERT.

ProteinBERT offers several advantages as a pLM. First, it has fewer parameters than other pLMs and does not require training on large datasets, facilitating pretraining on non-redundant datasets and avoiding biased pretraining on specific protein families. Consequently, fine-tuning requires less time and memory. Second, pretraining can utilize information from the GO to learn functional information about proteins.

### 2.3 Fine-tuning

We fine-tuned ProteinBERT to predict the resistance mechanism of each ARG (cf. Fig. 1). To predict one of the six resistance mechanism labels, we first added a fully connected layer and a softmax layer to the global representation output of ProteinBERT. We then trained and tested the model using a stratified 5-fold CV. Specifically, using the dataset divided as described in Section 2.1, we trained on one group and tested the model on the remaining one. This process was repeated five times and the average results were reported. For improvement of performance, we followed the training approach proposed by Brandes *et al.* (2022). See Supplementary Section S2.2 for the detailed fine-tuning procedure. Training and evaluation were conducted using two NVIDIA A100 Graphics Processing Units (GPUs; 80GB).

### 2.4 Comparison with baseline models

The performance of the proposed method was compared with that of LM-ARG, HMD-ARG, BLAST, and CARD-RGI. LM-ARG predicts the resistance mechanism by passing the embedding provided by ProtAlbert (Elnaggar *et al.* 2021), one of the pLMs, through the pooling and feed-forward layers (Ahmed *et al.* 2022). HMD-ARG is a resistance mechanism prediction model that uses a 6-layer CNN, developed by Li *et al.* (2021). Although they predicted only sequences predicted as ARG at Level 0, we predicted all input sequences of resistance mechanisms. BLAST (Altschul *et al.* 1990) was used to predict by retrieving similar sequences from the test set, using the training data as a reference. The resistance mechanism was estimated using the hit sequence with the lowest e-value. CARD-RGI is a BLAST-based model that predicts resistance mechanisms based on bit scores, using the CARD (Alcock *et al.* 2020, 2023) as a reference. In the predictions made by BLAST and CARD-RGI, sequences with no similar sequence hit in reference sequences were classified as “others.”

### 2.5 Evaluation metrics

We evaluated the performance of the proposed method by employing indicators of Accuracy, Precision, Recall, and *F*1-score. For Precision, Recall, and *F*1-score, we considered the prediction of each resistance mechanism as a binary classification problem, distinguishing between the focused mechanism and all other mechanisms. Based on this, we calculated the True Positive (TP), True Negative (TN), False Positive (FP), and False Negative (FN) values for each resistance mechanism. These metrics were then computed using the following equations:

$$\begin{array}{c} \text{Precision}=\frac{\text{TP}}{\text{TP}+\text{FP}},\;\;\text{Recall}=\frac{\text{TP}}{\text{TP}+\text{FN}}\\ F1-\text{score}=\frac{2\times \text{Precision}\times \text{Recall}}{\text{Precision}+\text{Recall}}. \end{array}$$

For Precision, Recall, and *F*1-score, we calculated the metrics for each resistance mechanism and then computed the macro average. Accuracy was calculated as the proportion of sequences for which the resistance mechanism was correctly predicted among all sequences in the dataset. We repeated this process for each iteration of 5-fold CV and then obtained the average of all iterations.

### 2.6 Attention analysis

Attention value is computed in ProteinBERT to incorporate local representations into global representations. The value is a per-amino acid value that is calculated as $z_{1},\ldots ,z_{L}$ in the following formula:

(1) $$z_{1},\ldots ,z_{L}=\text{softmax}\left({\left\{\frac{\langle q,k_{i}\rangle }{\sqrt{d_{\text{key}}}}\right\}}_{i=1}^{L}\right)$$

where $q\in R^{d_{\mathrm{query}}}$ and $k_{i}\in R^{d_{\mathrm{key}}}$ are the query and key vectors, respectively. The *q* and $k_{i}$ are used for incorporating the global and local representation into global attention. They are calculated as $q=\tanh(W_{q}x),k_{i}=\tanh(W_{k}s_{i})$ where *x* is the global representation, $s_{i}$ is the local representation, and $W_{k},W_{q}\in R^{d_{\mathrm{key}}\times d_{\mathrm{local}}}$ are the weight parameters.

To elucidate the biological significance of attention in the proposed method, we performed two analyses: (i) comparison with amino acid conservation (Section 2.6.1) and (ii) identification of functionally relevant domains where attention is concentrated (Section 2.6.2).

#### 2.6.1 Comparison with amino acid conservation

We examined the relationship between attention and a conservation score, which indicates the degree of conservation for each amino acid residue among the evolutionary-related bacteria. See Supplementary Section S2.3 for the details of computing the conservation score. First, we computed the Spearman correlation between attention and conservation scores. Second, we divided the residues into three groups according to their attention values, using the 33rd and 66th percentiles as thresholds. Finally, we assessed differences in the distribution of conservation scores between these groups using the Mann–Whitney *U* test (one-sided, greater) with Bonferroni correction (significance level *P* = .05/*N*, where *N* is the number of tests, *N* = 3).

#### 2.6.2 Identification of “attention-intensive regions” and their biological function

We identified sequence regions where attention is concentrated and named them “attention-intensive regions.” We used InterProScan (Jones *et al.* 2014, Paysan-Lafosse *et al.* 2023) to identify specific sequence regions corresponding to families, domains, and functional sites conserved in the analyzed sequences. We then compared the distribution of attention in these regions with the whole sequence using the Mann–Whitney *U* test (one-sided, greater), and the significant regions are defined as “attention-intensive regions.”

We attempted to clarify the biological function of attention-intensive regions. Identified sequence regions were annotated with GO terms by InterProScan (Jones *et al.* 2014). We performed GO enrichment analysis to infer the function of the attention-intensive sequence regions based on GO (Gene Ontology Consortium *et al.* 2023). We used Fisher’s exact test (one-sided, greater) to determine whether each GO term was significantly enriched in attention-intensive regions. We restricted the sequence regions used in our analysis to those annotated with at least one GO term.

In the above, to determine significance, we applied the Bonferroni correction in all analyses (significance level *P* = .05/*N*, where *N* is the number of tests).

## 3 Results

### 3.1 Performance comparison

**Figure 2.**
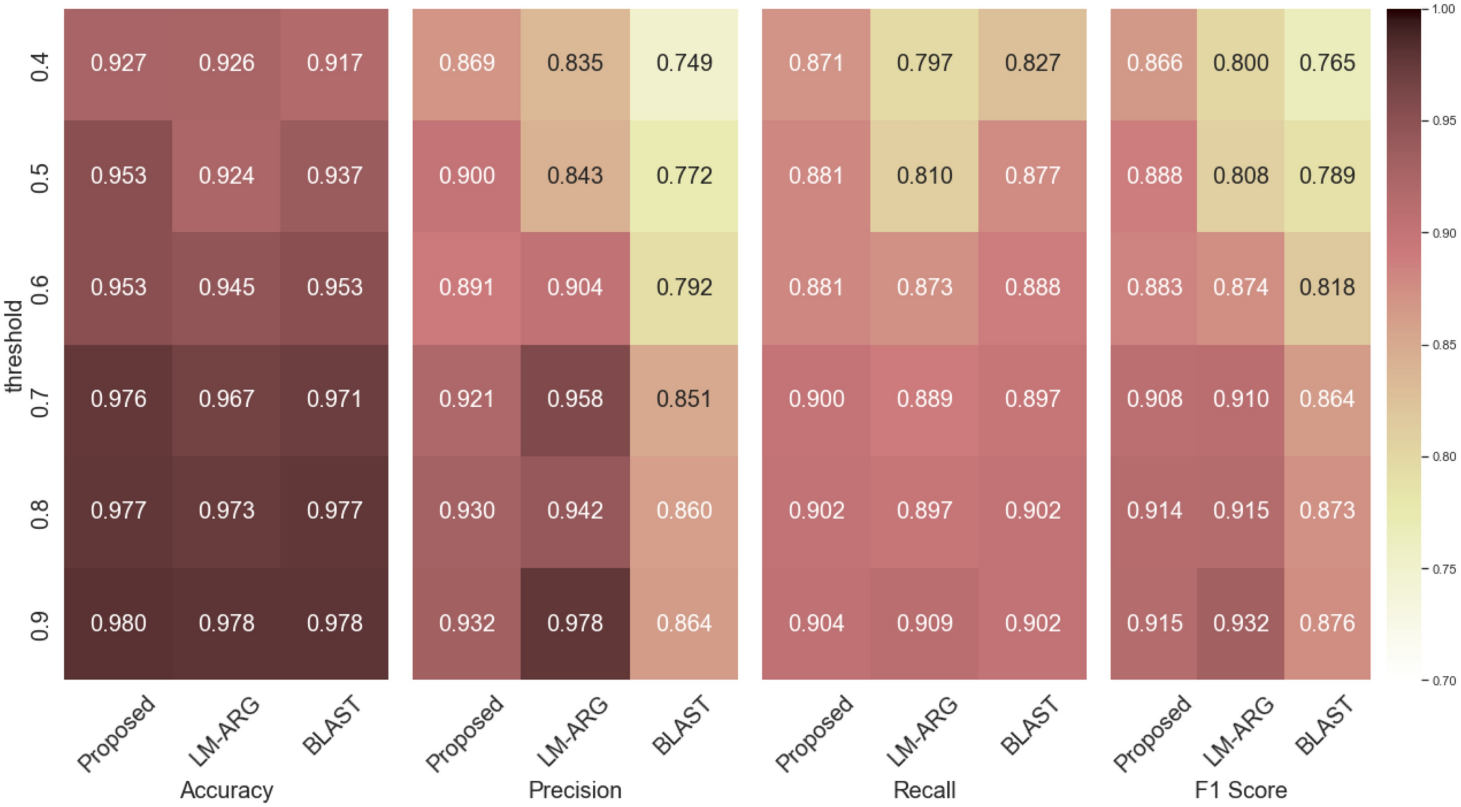
Performance comparison of the proposed method, LM-ARG, and BLAST low homology datasets generated with varying sequence identity thresholds (see Section 2.1.2 for dataset generation details) with respect to Accuracy, Precision, Recall, and *F*1-score (see Section 2.5 for metric definitions). The *y*-axis indicates the sequence identity threshold used to create each low homology dataset, ranging from 0.4 to 0.9 in increments of 0.1.

First, the performance of the proposed method was compared to that of LM-ARG, HMD-ARG, BLASTP, and CARD-RGI (cf. Section 2.4) using the HMD-ARG DB. The performance indicators of the proposed method, namely Accuracy, Precision, Recall, and *F*1-score, achieved 0.999, 0.943, 0.934, and 0.937 (Table 1, top). On the other hand, the existing methods with the best performance, LM-ARG and BLAST, were 0.980, 0.929, 0.902, 0.911, and 0.987, 0.910, 0.933, 0.919, respectively. Additionally, the detailed performances for each resistance mechanism are also shown in Supplementary Fig. S3. (The “other” resistance mechanism was excluded due to the small number of sequences.) All the methods achieved better performances for the resistance mechanisms with a large number of sequences than for those with a small number of sequences. In summary, when trained on the HMD-ARG DB, the proposed model performed slightly better than the best-performing existing methods, BLAST and LM-ARG. Next, we performed a performance comparison using the low homology dataset (Table 1, bottom and Fig. 2). The proposed method achieved an Accuracy of 0.927, Precision of 0.869, Recall of 0.871, and *F*1-score of 0.866, outperforming the other methods, which achieved values of 0.926, 0.835, 0.797, 0.800 (LM-ARG), and 0.917, 0.749, 0.827, 0.765 (BLAST). Furthermore, the proposed method was compared with LM-ARG and BLAST, which had performed well on the HMD-ARG DB, on datasets with sequence identity thresholds ranging from 0.4 to 0.9. While the proposed method performed comparably or slightly below LM-ARG and BLAST on datasets with sequence identity thresholds of 0.6–0.9, it notably outperformed them on datasets with thresholds of 0.4 and 0.5 (Fig. 2). The performances for each mechanism are shown in Supplementary Fig. S4. These results suggest that the proposed model accurately predicts resistance even for ARGs with low sequence similarity to known ARGs.

**Table 1.** The comparison of performances of two datasets: HMD-ARG DB and low-homology dataset.^a^

	Dataset	Accuracy	Precision	Recall	*F*1-score
Proposed		**0.999**	**0.943**	**0.934**	**0.937**
LM-ARG		0.980	0.929	0.902	0.911
HMD-ARG	HMD-ARG	0.968	0.920	0.871	0.889
BLAST	DB	0.987	0.910	0.933	0.919
CARD-RGI		0.910	0.803	0.885	0.784
Proposed	Low	**0.927**	**0.869**	**0.871**	**0.866**
LM-ARG	Homology	0.926	0.835	0.797	0.800
BLAST	Dataset	0.917	0.749	0.827	0.765

a The bold values indicate the highest value for a combination of metric and dataset.

To confirm the usefulness of the proposed method, we carried out two further detailed evaluations. First, we compared the time taken to train the model and predict with existing machine learning models. We found that the proposed method is more efficient than LM-ARG (Supplementary Table S1). Note that we could not compare with HMD-ARG because we only had trained ones. Second, to assess the contribution of fine-tuning, we evaluated a model without fine-tuning. This model was created by freezing the parameters of ProteinBERT during training. The proposed method with fine-tuning was comparable to or slightly better than the model without fine-tuning, suggesting the effectiveness of fine-tuning (Supplementary Figs S2–S4).

### 3.2 Attention analysis

We selected four ARGs for attention analysis based on specific criteria. For macB and tetW, we first identified the two most prevalent families within each resistance mechanism. We then chose sequences that had at least one significant domain and contained a high number of domains (five or more). In the case of rpoB, our initial intention was to analyze this sequence alongside information on point mutations, so we selected a sequence with available point mutation data. Lastly, blaOXA-114s was chosen because beta-lactamases are highly prevalent and play a crucial role in the antibiotic inactivation mechanism. Supplementary Table S2 provides detailed information on the selected sequences, including the ARG family, HMD-ARG DB accession, resistance mechanism, and description of the ARGs.

#### 3.2.1 Relationship between attention and amino acid conservation

**Figure 3.**
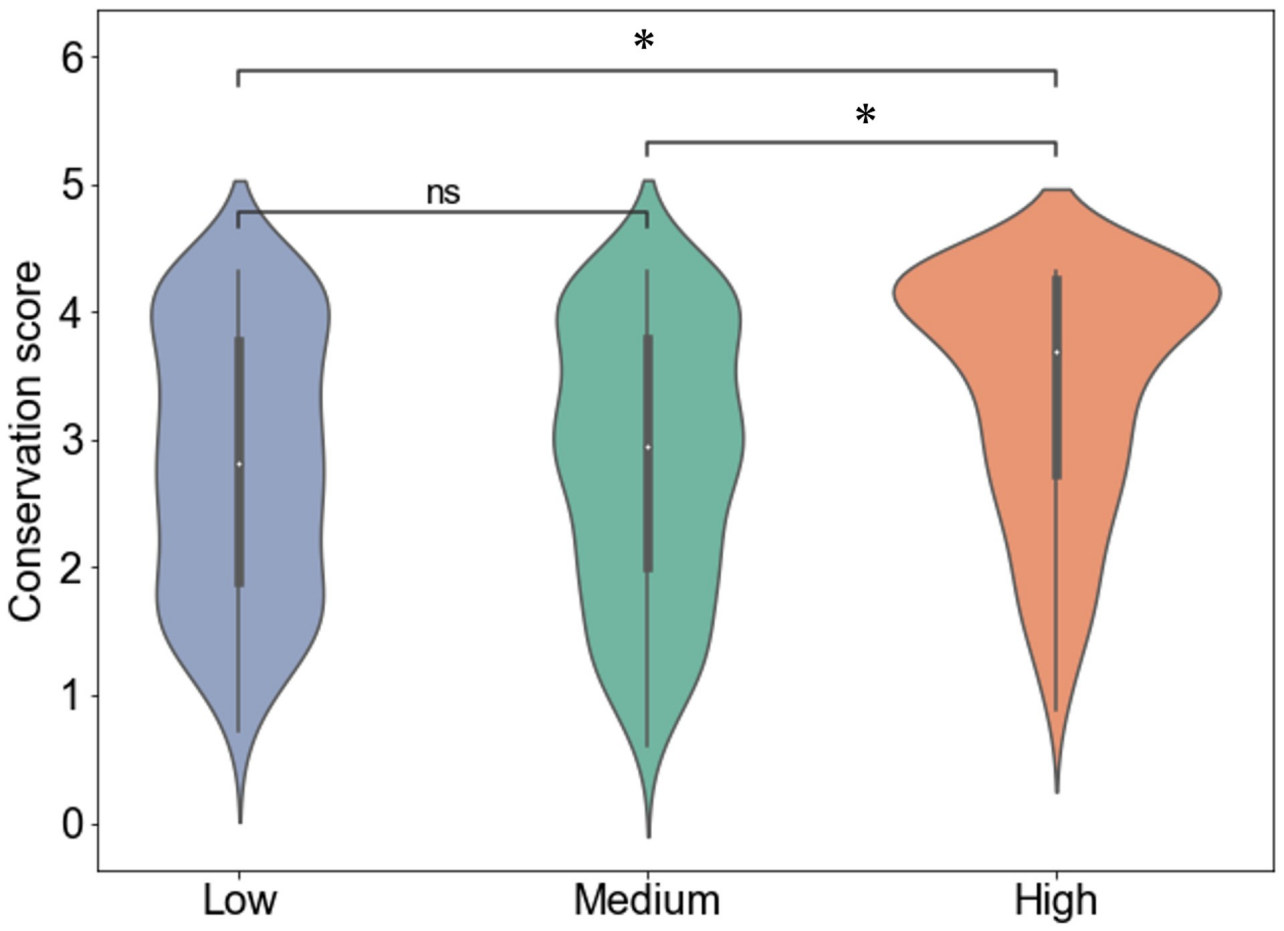
Relationship between attention values and amino acid conservation scores in tetW. Attention values were divided into three groups: Low (attention < 33rd percentile), Medium (33rd percentile < attention < 66th percentile), and High (attention > 66th percentile). The distribution of conservation scores was compared among these groups using the Mann–Whitney *U* test with Bonferroni correction (significance level: *P* < .05/3). “ns” indicates no significant difference (0.05/3 < *P* < 1), while “*” indicates a highly significant difference (*P* < .05/3) between the conservation score distributions.

Previous studies have shown that in pLMs predicting solubility, highly conserved regions contribute to the prediction (Thumuluri *et al.* 2022). Motivated by this finding, we compared the attention values and conservation of amino acids for each residue in tetW, and found a significant correlation as in Supplementary Fig. S6a (Spearman correlation coefficient 0.258, *P* = 3.37e−11). Furthermore, amino acid residues were classified into three groups based on attention values: low, medium, and high, using the 33rd and 66th percentiles as thresholds (33rd percentile = −2.67e−4, 66th percentile = −7.94e−5). The Mann–Whitney *U* test (significance level *P* < .05/*N*, where *N* is the number of tests, i.e. *N* = 3) was then used to compare the conservation score distributions between these groups. The results showed that the distribution of conservation scores for the “high” group was significantly greater than the distributions for the “low” and “medium” groups (Medium versus High: *P* = 5.05e−09, Low versus High: *P* = 3.20e−09). However, no significant differences were found between the “low” and “medium” groups (Fig. 3, *P* = .426). These findings suggest that attention in the proposed model recognizes highly conserved amino acid residues, but it cannot be concluded that amino acid residues with low conservation scores necessarily have low attention values. Additionally, the analysis results of rpoB, blaOXA-114s, and macB are given in Supplementary Figs S5 and S6. The analysis for rpoB and macB showed that, similar to tetW, the amino acids in the “High” group had significantly higher conservation scores than the “Low” and “Medium” groups. In blaOXA-114s, there were no significant differences in the distribution of conservation scores among all groups. On the other hand, Supplementary Fig. S6c shows that the 13 amino acid residues with the highest attention values in blaOXA-114s have high conservation scores. The analysis results of these three ARGs suggest that several ARGs shared the ability of attention to identify conserved residues. This may be because pLMs have acquired evolutionary information about proteins through pretraining and fine-tuning (Ding and Steinhardt 2024, Zhang *et al.* 2024).

#### 3.2.2 Biological functions of attention-intensive sequence regions

Previous studies have shown that in BERT models pretrained on antibody sequences, attention is concentrated on amino acid residues located in antigen-binding sites (Leem *et al.* 2022). We hypothesized that the attention in our proposed model would exhibit similar characteristics. In the following, we focus on four ARGs listed in Supplementary Table S2 for attention analysis, based on attention-intensive regions (cf. Section 2.6.2). Table 2 (top) shows four attention-intensive regions in rpoB. Notably, domain PF04565, which represents RNA polymerase beta subunit domain 3, is known as the fork domain and is involved in binding to the target antibiotic rifampin (Alifano *et al.* 2015). On the other hand, Table 2 (bottom) presents the attention-intensive regions in tetW, with significantly high attention values observed in the guanosine-5'-triphosphate (GTP)-binding domain. GTP-binding has been shown to play a crucial role in tetW-mediated antibiotic resistance (Chopra and Roberts 2001). This finding is supported by the observation that mutations in the GTP-binding site altered the affinity for the target antibiotic (Chopra and Roberts 2001). These results demonstrate that attention recognizes the antibiotic binding site in rpoB and the site that contributes to antibiotic resistance in tetW.

**Table 2.** Attention-intensive regions identified by InterProScan for rpoB and tetW.^a^

AMR family	Accession	Database	Signature description	*P*-value
rpoB	G3DSA:3.90.1100.10	Gene3D	–	2.49e−11
	G3DSA:3.90.1800.10	Gene3D	RNA polymerase alpha subunit dimerization domain	4.86e−10
	G3DSA:3.90.1800.10:FF:000001	FunFam	DNA-directed RNA polymerase subunit beta	4.86e−10
	PF04565	Pfam	RNA polymerase Rpb2, domain 3	1.94e−14
tetW	PR00315	PRINTS	GTP-binding elongation factor signature	1.33e−08
	PTHR43261	PANTHER	Translation elongation factor G-related	4.37e−05
	TIGR00231	NCBIfam	JCVI: small GTP-binding protein domain	1.12e−04

a The table lists the significantly enriched InterPro signatures (*P*-value < .05 after Bonferroni correction) in the attention-intensive regions of each ARG. The columns show the AMR family, the accession number of the InterPro signature, the database from which the signature was derived, a brief description of the signature, and the *P*-value indicating the significance of the attention enrichment in the corresponding region.

**Figure 4.**
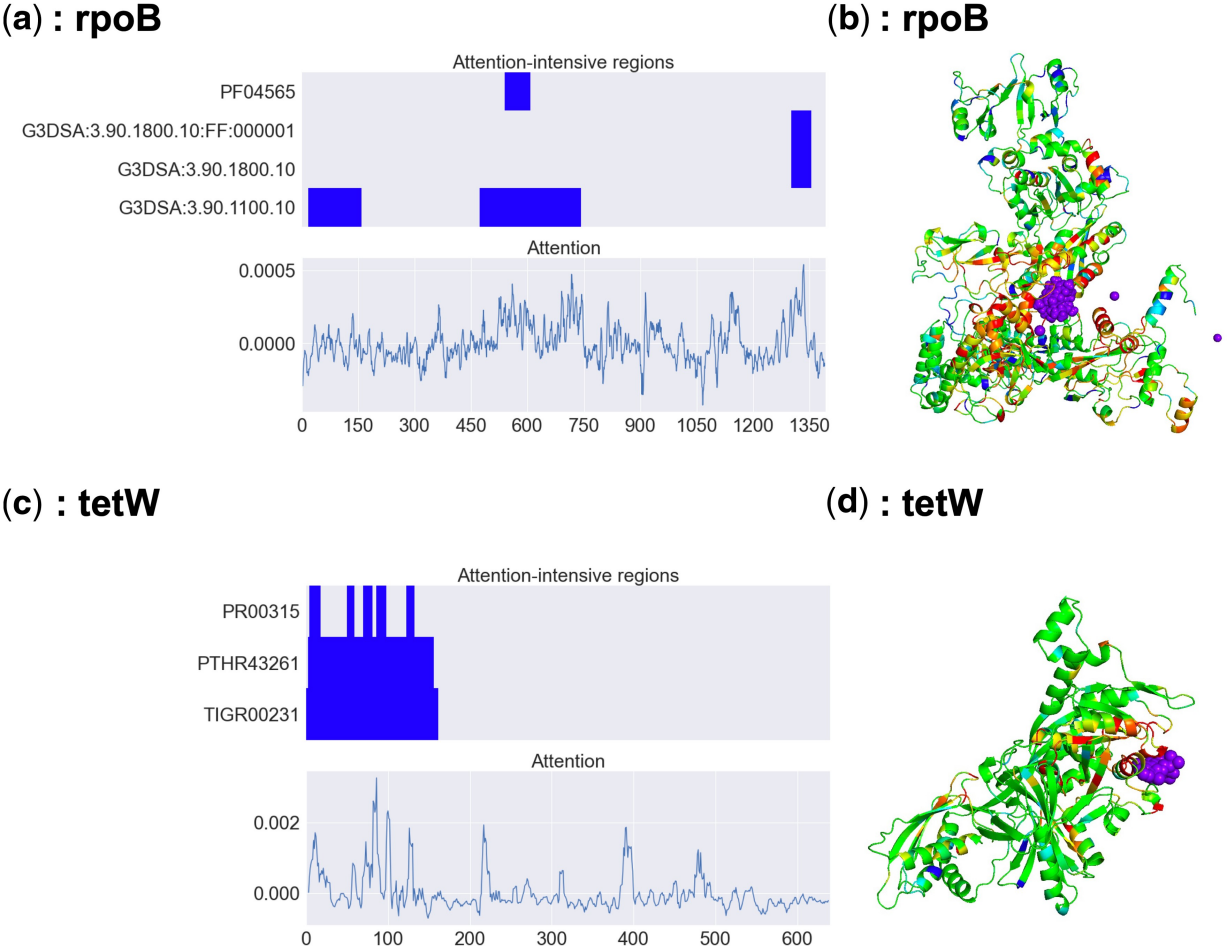
Attention analysis of rpoB and tetW. (a, c) Top: Attention-intensive regions (cf. Section 3.2.2) for InterPro-identified regions in rpoB and tetW, respectively. Bottom: Smoothed attention values were obtained by taking the average of five residues, with the target residue in the middle. The two graphs share an *x*-axis indicating the amino acid position. (b, d) Attention values mapped onto the 3D structures of rpoB (AlphaFold DB ID: AF-B4RQW2-F1) and tetW (AlphaFold DB ID: AF-A3R795-F1), respectively. Residues with high attention are colored red, while those with low attention are colored blue. The purple crystal structures represent rifampin and GTP, which were obtained from the crystal structures of homologous sequences (Protein Data Bank IDs: 4kmu and 2hcj, respectively).

Next, we conducted more detailed attention analyses (Fig. 4) for rpoB and tetW. Figure 4a and c shows the corresponding positions (top) and attention values (bottom) of the rpoB and tetW domains, respectively. These figures confirm that the regions where significant differences in the distribution of attention were identified show specifically high attention values. In addition, Fig. 4b and d illustrate the attention values mapped onto the predicted three-dimensional structures of rpoB and tetW using AlphaFold (Jumper *et al.* 2021, Varadi *et al.* 2022). Amino acids with high attention values, highlighted in red, are clustered around the purple binding molecules (rifampin in Fig. 4b, GTP in Fig. 4d) in three-dimensional spaces. All the figures also suggest that attention is concentrated on the rifampin or GTP binding site.

Supplementary Figure S7 and Supplementary Table S4 indicate the results for BlaOXA-114a, suggesting that the class-D active site that hydrolyzes the target beta-lactam has higher attention values (Bauernfeind 1986). Supplementary Figure S8 and Supplementary Table S4 indicate the results for macB, an adenosine triphosphate (ATP) binding cassette (ABC) transporter commonly found in antibiotic efflux, suggesting that significant attention concentrations are observed at the conserved site of the ABC transporter, the ATP-binding site. These results suggest that attention in the proposed method focuses on conserved regions that contribute to resistance.

**Figure 5.**
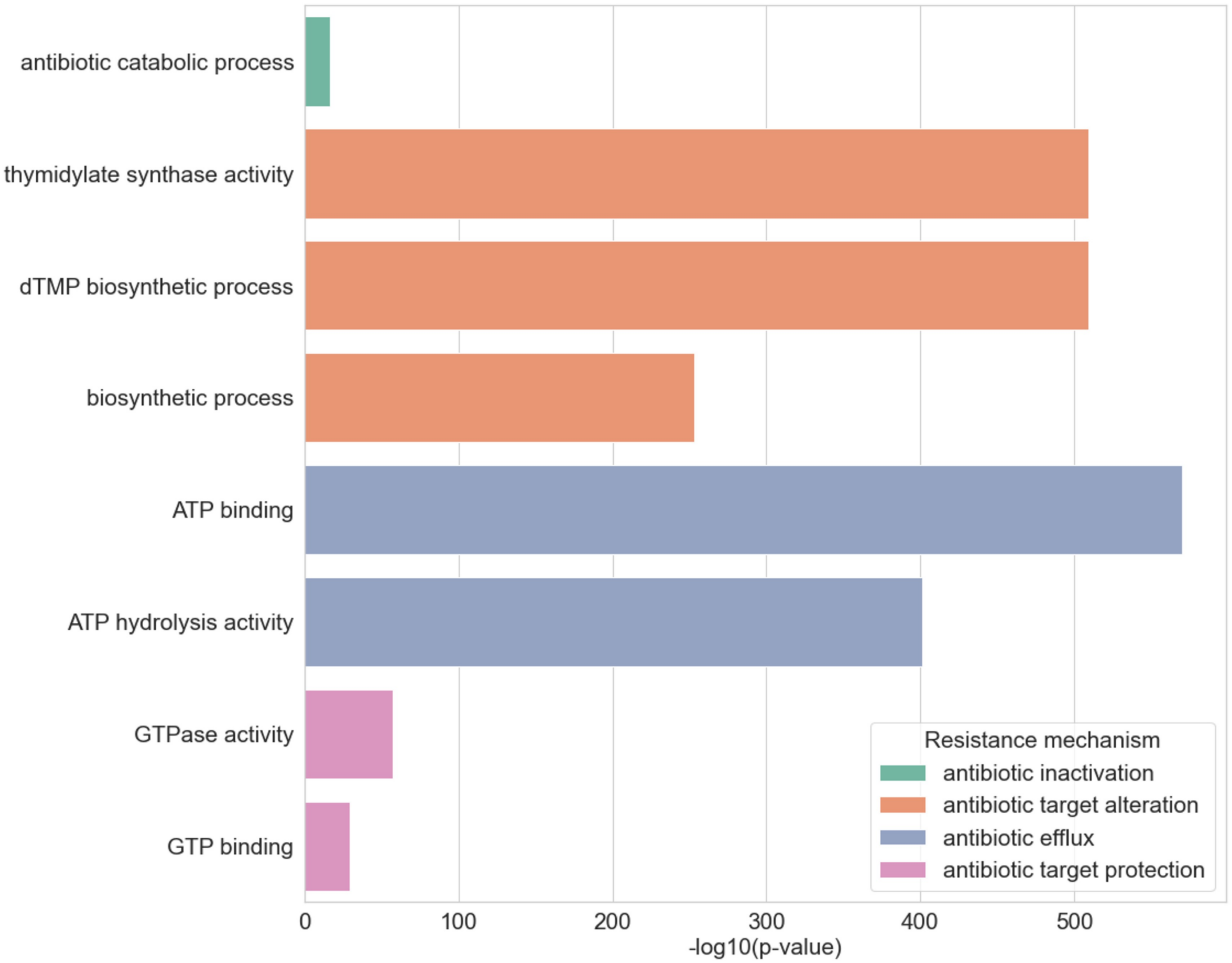
GO terms enriched in attention-intensive regions within ARG sequences belonging to each resistance mechanism. GO terms annotated in over 150 attention-intensive regions were selected for visualization from all significantly enriched GO terms using Fisher’s exact test. The *x*-axis represents the −log10 (*P*-value) for each GO term, while the *y*-axis lists the GO terms. *P*-values were corrected for multiple testing using the Bonferroni method. The color of the bars indicates the type of resistance mechanism.

Finally, we investigated whether the attention characteristics observed in individual ARGs are common across other sequences in the HMD-ARG DB. We identified sequence regions where attention was significantly concentrated and performed GO enrichment analysis on these attention-intensive regions using Fisher’s exact test with Bonferroni correction for multiple testing. The results, as shown in Fig. 5, revealed that GO terms such as “GTP binding” and “GTPase activity” were enriched in antibiotic target protection, while “ATP binding” and “ATP hydrolysis activity” were enriched in antibiotic target alteration. These findings suggest that the attention patterns observed in tetW and macB are consistent across other sequences. Furthermore, the enrichment of “antibiotic catabolic process” in “antibiotic inactivation” indicates that, similar to blaOXA-114s and rpoB, attention can identify sequence regions that bind and interact with the target antibiotic. Collectively, these results demonstrate that attention in our model recognizes interaction sites and highly conserved regions associated with target antibiotics across a wide range of ARGs in the HMD-ARG DB.

## 4 Discussion

In this study, we developed a novel computational approach to predict resistance mechanisms from ARG sequences using a pLM. Although the proposed model is similar to LM-ARG in its pLM-based model for predicting resistance mechanisms, they differ in three key ways. First, LM-ARG is a model whose classifier is trained by ProtAlbert embedding, whereas the proposed model is a fine-tuned ProteinBERT. Second, the proposed model performed slightly better or comparably for ARGs with high similarity to training data, and much better for ARGs with low similarity. Third, the proposed model provided faster training and prediction compared to LM-ARG.

Moreover, the attention analysis revealed that our model is interpretable and considers biologically relevant features, such as amino acid conservation and target binding sites when making predictions (Section 3.2). The attention analysis also suggests that our model may have the potential to recognize tertiary structures and point mutations, but further investigation is required to confirm these hypotheses. This interpretability for predicting resistance mechanisms is another advantage not present in existing methods, including LM-ARG, and can provide valuable insights into the molecular basis of antibiotic resistance.

It is important to acknowledge the limitations of the current study, particularly in terms of data collection and analysis methods. Firstly, the reliability of the annotation methods used for training and evaluating datasets is questionable due to the lack of a unified definition and the scarcity of databases containing information on the nature of ARGs, such as resistance mechanisms. The inconsistency in annotation methods, with some based on the antimicrobial resistance (AMR) family and others relying on the predicted results of existing methods, further compounds this issue. The credibility of these methods remains uncertain, and the development of a more accurate and uniform database would greatly assist in predicting and analyzing resistance mechanisms. Secondly, our analysis may have overlooked unknown attention-intensive regions or features. By relying on known databases, we risk overlooking attention-intensive regions not present in them, including unknown functional sites related to resistance mechanisms. Additionally, we only compared attention and amino acid conservation for four ARGs, limiting our understanding. To gain a more accurate understanding, a comprehensive analysis of the most ARGs in the datasets is necessary. Improved methods for analyzing attention could potentially reveal important sequence regions and features that have not yet been discovered, providing deeper insights into the molecular basis of antibiotic resistance. Thirdly, it is worth making a more rigorous comparison with LM-ARG. We have compared the proposed method with fine-tuning with LM-ARG, a method without fine-tuning, and concluded that the proposed method is slightly better. Furthermore, it would be worthwhile to investigate whether fine-tuning improves the performance of LM-ARG and outperforms the proposed method. Despite these limitations, our study demonstrates the potential of using pretrained pLMs for predicting and understanding ARG resistance mechanisms, with several promising applications. The attention analysis results, which identify conserved regions and binding sites associated with antibiotic resistance, could guide researchers in designing novel antibiotics that target these specific areas, potentially leading to the development of more effective drugs that can overcome existing resistance mechanisms. Furthermore, by leveraging the model’s ability to accurately predict resistance mechanisms, even for ARGs with low sequence similarity to known sequences, it may be possible to develop rapid diagnostic tools for the early detection and monitoring of antibiotic-resistant bacteria. These tools could help healthcare professionals identify resistant strains more quickly, allowing for the timely implementation of appropriate treatment strategies and infection control measures, ultimately contributing to the global effort in combating antibiotic resistance.

## Supplementary Material

btae550_Supplementary_Data
